# Heparin-free veno-venous extracorporeal membrane oxygenation as a bridge therapy for airway interventions in malignant central airway obstruction: Case series and literature review

**DOI:** 10.1097/MD.0000000000050498

**Published:** 2026-09-04

**Authors:** Siyu Yang, Zhenshun Cheng, Qing Liu, Lan Ni, Chaojie Wei

**Affiliations:** a Department of Pulmonary and Critical Care Medicine, Zhongnan Hospital of Wuhan University, Wuhan, China.

**Keywords:** anticoagulation, bronchoscopy, malignant airway obstruction, respiratory failure, veno-venous extracorporeal membrane oxygenation

## Abstract

**Rationale:**

Malignant central airway obstruction (MCAO) represents a life-threatening oncologic emergency that can lead to rapid respiratory decompensation and death. Traditional management with mechanical ventilation may be inadequate or risky when severe airway narrowing or complete obstruction exists. Veno-venous extracorporeal membrane oxygenation (VV-ECMO) offers an alternative method of gas exchange that can support patients during high-risk airway interventions. This article presents 3 cases and reviews existing literature to evaluate the role of VV-ECMO as a bridge therapy for airway procedures in patients with MCAO.

**Patient concerns:**

All 3 patients presented with symptomatic airway stenosis secondary to malignant pulmonary or metastatic tumors, and conventional tracheal intubation failed to maintain adequate oxygenation and ventilation, necessitating urgent advanced respiratory support.

**Diagnoses:**

All 3 patients were diagnosed with respiratory failure secondary to bronchial obstruction caused by primary or secondary malignant lung tumors.

**Interventions:**

All patients were initiated on heparin-free VV-ECMO to establish stable extracorporeal gas exchange, followed by definitive interventional therapy via flexible bronchoscopy, including tumor debulking and/or airway stenting.

**Outcomes:**

Immediate improvement in oxygenation was achieved in all 3 patients following ECMO-supported intervention.

**Lessons:**

VV-ECMO serves as a viable bridge to definitive airway interventions in carefully selected patients with MCAO. Anticoagulation management requires careful balance, with a no-heparin protocol appearing safe for brief ECMO runs. Multidisciplinary collaboration is essential for optimal outcomes in these complex cases.

## 1. Introduction

Malignant central airway obstruction (MCAO) represents one of the most critical emergencies in thoracic oncology, often presenting with acute respiratory distress that can rapidly progress to fatal asphyxia.^[1,2]^ The incidence of this complication has been rising in parallel with the increasing population of cancer patients, particularly those with lung cancer, esophageal cancer, and metastatic disease to the mediastinum.^[3,4]^ When tumors directly invade the tracheobronchial tree or cause extrinsic compression of central airways, the resulting narrowing can critically compromise ventilation and gas exchange, creating a life-threatening scenario that demands immediate intervention. Surgical resection and reconstruction remain the gold standard for curative intent in select resectable tumors^[5]^; however, the majority of patients with MCAO present with advanced, unresectable disease or are medically unfit for surgery due to comorbidities or debilitation. For these patients, palliative interventions – including bronchoscopic tumor debulking, airway stenting, or emergency radiation therapy – are the mainstay of care.^[6–8]^ However, performing these interventions in patients with severe or near-complete airway obstruction presents considerable challenges. Conventional mechanical ventilation may be inadequate or even dangerous in these circumstances, as positive pressure can be difficult to deliver through critically narrowed airways, and airway instrumentation during bronchoscopy may precipitate complete obstruction. This therapeutic dilemma has led clinicians to explore alternative respiratory support strategies that can maintain oxygenation and ventilation while allowing definitive airway procedures to be performed safely.

Extracorporeal membrane oxygenation (ECMO), specifically the veno-venous (VV) configuration, has emerged as a potential solution to this problem.^[9,10]^ By providing extracorporeal gas exchange independently of native lung function, VV-ECMO can maintain adequate oxygenation and carbon dioxide removal even during complete airway obstruction or when the airway must be shared with procedural instruments.^[11,12]^ This capability makes ECMO particularly valuable as a bridge to definitive therapy – a temporary support system that stabilizes the patient during high-risk airway interventions. Despite its theoretical utility, the use of VV-ECMO in oncology patients remains controversial. Historically, active malignancy was considered a relative contraindication to ECMO, driven by concerns about poor long-term prognosis, increased bleeding risk (especially during invasive procedures or biopsies), and heightened thrombosis risk associated with both malignancy and extracorporeal support.^[13,14]^ The role of VV-ECMO in cancer-related respiratory failure thus remains incompletely defined, with indications requiring rigorous multidisciplinary team (MDT) assessment.^[15]^ However, emerging evidence from case series and small cohort studies suggests that carefully selected cancer patients – particularly those with reversible causes of respiratory failure such as MCAO – can derive meaningful benefit from VV-ECMO support.^[4,16]^

To further elucidate the role of VV-ECMO in this challenging clinical scenario, we present 3 cases of MCAO managed with heparin-free VV-ECMO as a bridge to bronchoscopic intervention. We complement these cases with a review of contemporary literature, focusing on ECMO utilization for airway obstruction, anticoagulation strategies, and patient selection. Our goal is to provide clinical insights into optimizing outcomes for patients with MCAO and to reinforce the value of a multidisciplinary approach to this life-threatening condition.

## 2. Case reports

### 2.1. Case 1

A 49-year-old male with no significant past medical history presented with 1 month of progressive dyspnea and was diagnosed with squamous cell carcinoma of the lung. Initial management included placement of a right bronchial stent to address tumor-related bronchial obstruction. Despite this intervention, he returned 1 month later with recurrent severe dyspnea.

**Figure 1.**
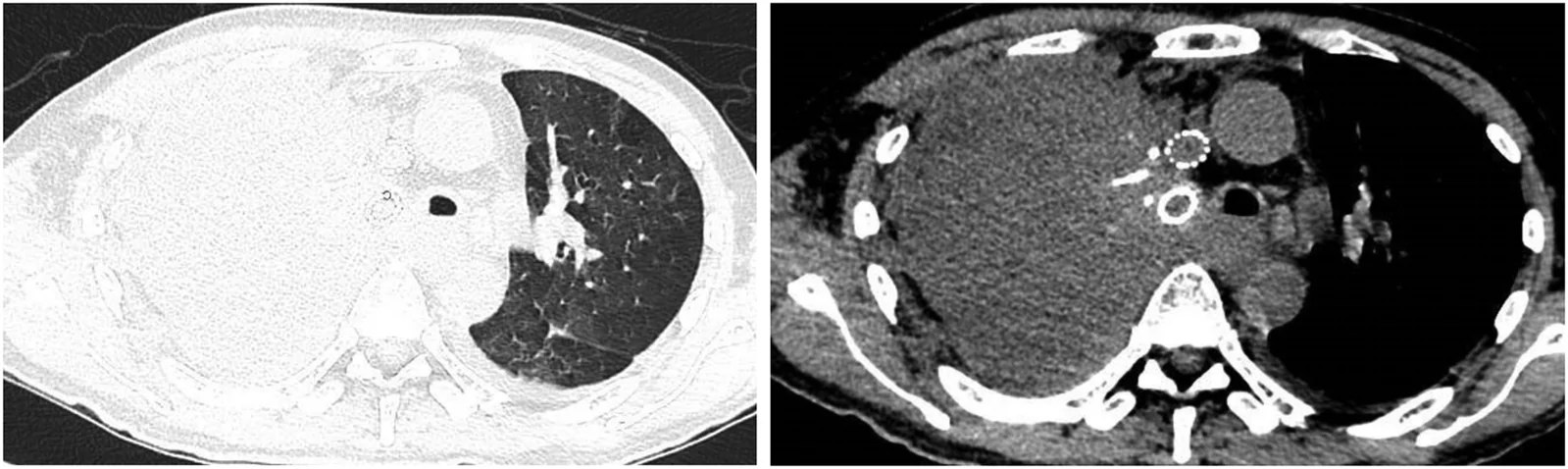
Right lung consolidation. Neoplastic lesions were identified within the right main bronchial stent, causing its obstruction.

**Figure 2.**
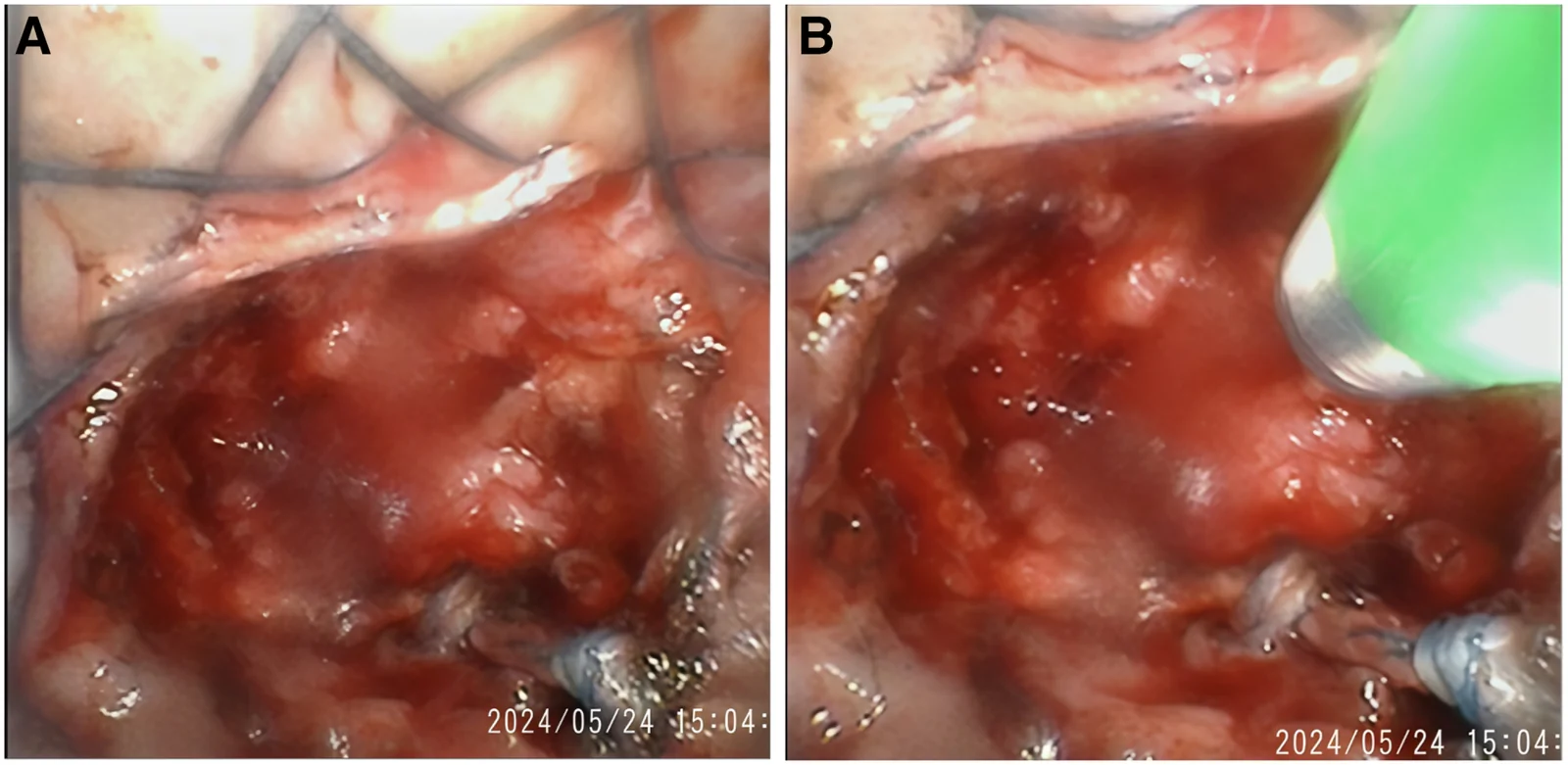
(A) A neoplasm was identified at the distal end of the right main bronchus, obstructing the lumen. (B) Electrosection was performed on the neoplasm.

On presentation, his oxygen saturation was 82% on room air, requiring immediate intubation and mechanical ventilation. Chest computed tomography (CT) revealed left lung pneumonia, right lung consolidation, and tumor regrowth obstructing the previously placed right main bronchus stent (Fig. 1). Bronchoscopy confirmed near-complete obstruction of the stent lumen by friable tumor tissue (Fig. 2A). Despite maximal ventilator support with 100% FiO_2_ and high PEEP, his oxygenation deteriorated with a PaO_2_/FiO_2_ ratio falling below 100 mm Hg.

Given the refractory hypoxemia and the need for definitive airway intervention, an MDT consisting of intensivists, interventional pulmonologists, and oncologists recommended initiating heparin-free VV-ECMO as bridge therapy. Cannulation was performed via the right femoral vein (drainage) and right internal jugular vein (return). Once ECMO flow was established and oxygenation stabilized (PaO_2_/FiO_2_ > 200 mm Hg), the patient underwent bronchoscopic tumor debulking using electrocautery (Fig. 2B). The procedure was performed successfully without the need for mechanical ventilation during the intervention. Total ECMO run time was 23 hours.

The patient was successfully weaned from ECMO on postoperative day 1 and extubated shortly thereafter, with no ECMO-related complications (e.g., thrombosis, bleeding, or cannulation site issues) observed. Unfortunately, 1 month after discharge, he developed progressive pulmonary infection and septic shock in the context of widely metastatic disease – an expected progression of his advanced malignancy – and ultimately expired. Despite this unfortunate long-term outcome, VV-ECMO effectively addressed the immediate life-threatening MCAO, restored airway patency, and provided meaningful symptomatic relief.

### 2.2. Case 2

A 63-year-old male with a history of esophageal carcinoma treated with curative-intent surgical resection 3 years prior presented to the emergency department with acute-onset severe dyspnea and hypoxemia. Initial assessment revealed oxygen saturation of 78% on 15 L/min high-flow nasal cannula, with bilateral wheezing and decreased breath sounds over the left hemithorax.

**Figure 3.**
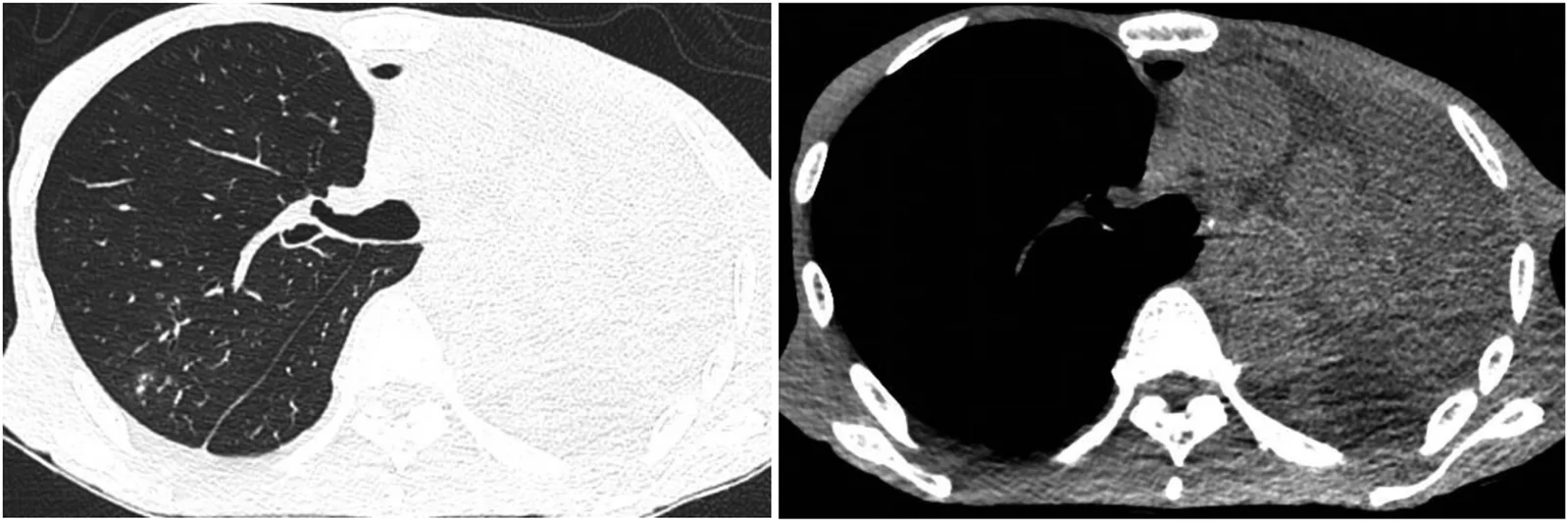
Left main bronchial occlusion, left lung atelectasis, and left pleural effusion.

**Figure 4.**
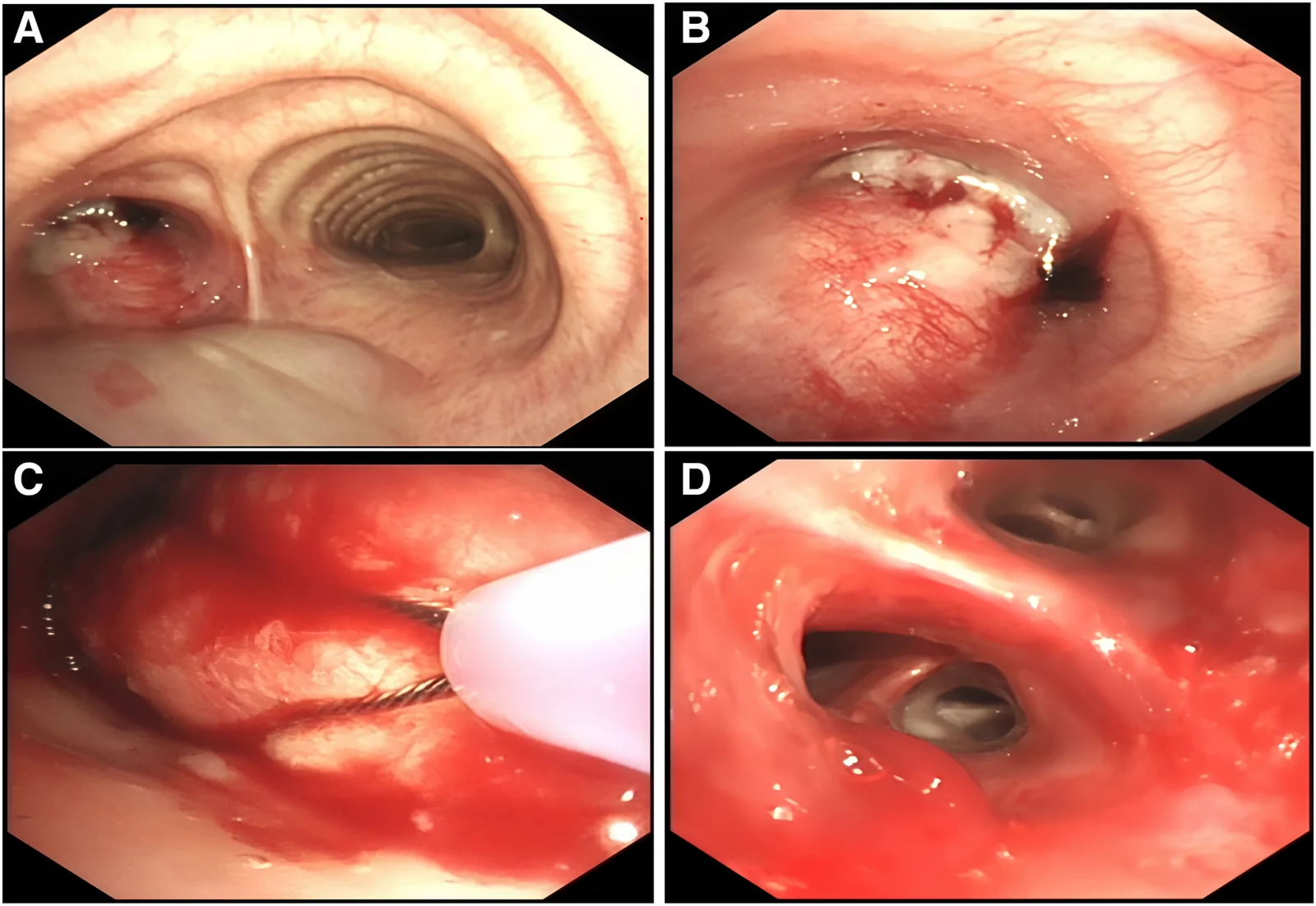
(A and B) A neoplasm at the orifice of the left main bronchus. (C) Endoscopic snare resection and ligation were performed. (D) The lumen of the left main bronchus was patent after the procedure.

Chest CT demonstrated a left hilar mass causing complete occlusion of the left main bronchus, associated left lung collapse, and a large left-sided pleural effusion (Fig. 3). Bronchoscopic evaluation confirmed malignant obstruction of the left main bronchus, with histopathological analysis consistent with metastatic esophageal carcinoma – representing a distant recurrence of his primary disease (Fig. 4A and B). The patient was emergently intubated due to worsening respiratory distress, but despite aggressive mechanical ventilation, therapeutic thoracentesis, and broad-spectrum antibiotics for concurrent pneumonia, his oxygenation continued to deteriorate, with a PaO_2_/FiO_2_ ratio of approximately 50 mm Hg – indicating life-threatening refractory hypoxemia.

The MDT convened and determined that VV-ECMO was the only viable option to stabilize the patient for definitive airway intervention. A heparin-free protocol was implemented: the ECMO circuit was primed with heparinized saline (5 U/mL) to mitigate circuit thrombosis, but no systemic heparin was administered during the ECMO run. Femoral–jugular cannulation was performed, and ECMO flow was titrated to 3.5 to 4.0 L/min, resulting in immediate stabilization of oxygenation (PaO_2_/FiO_2_ > 250 mm Hg).

On the same day as ECMO initiation, the patient underwent bronchoscopic intervention: electrocautery snare was used to debulk the obstructing metastatic tumor (Fig. 4C and D), followed by placement of a covered metal stent in the left main bronchus to maintain airway patency. Oxygenation remained stable throughout the procedure, with no intraprocedural complications. ECMO was weaned and discontinued on postoperative day 1, and the patient was extubated later that afternoon, with marked improvement in respiratory status.

One week later, the patient underwent revision bronchoscopy with rigid bronchoscopy, during which the initial stent was replaced with a customized Y-shaped covered metal stent to better conform to the airway anatomy, along with additional tumor debulking. He was discharged home 2 weeks after the index procedure, with normal oxygenation on room air and no respiratory symptoms. At 6-month follow-up, he remained asymptomatic with no evidence of recurrent airway obstruction, demonstrating the durable clinical benefit of ECMO-supported definitive airway intervention.

### 2.3. Case 3

**Figure 5.**
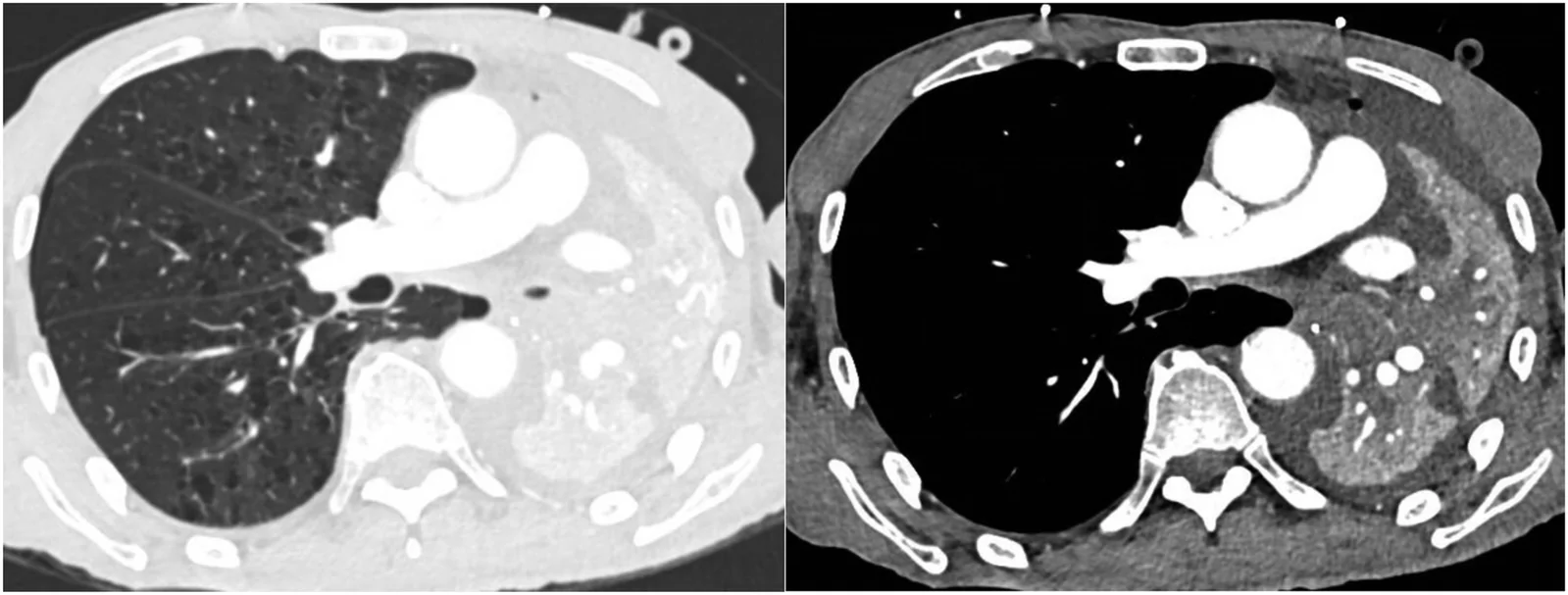
Left lung inflammation complicated with atelectasis, left bronchial occlusion, and enlarged hilum. Contrast-enhanced imaging suggested a neoplastic process.

A 72-year-old male with a history of type 2 diabetes mellitus presented with a 3-week history of progressive nonproductive cough, exertional dyspnea, and left-sided chest discomfort. On initial evaluation in the emergency department, he was found to have severe hypoxemia, with arterial blood gas analysis revealing a PaO_2_ of 22 mm Hg on room air – consistent with life-threatening hypoxemic respiratory failure. Emergency chest CT demonstrated a large mass completely occluding the left main bronchus, with associated left lung collapse and mediastinal shift (Fig. 5).

The patient was emergently intubated, but given the profound hypoxemia and concern for tenuous airway patency (with risk of complete obstruction during bronchoscopy), heparin-free VV-ECMO was initiated immediately after intubation. Femoral–jugular cannulation was performed, and ECMO flow was set at 3.2 L/min, resulting in rapid improvement in oxygenation (PaO_2_/FiO_2_ > 200 mm Hg).

**Figure 6.**
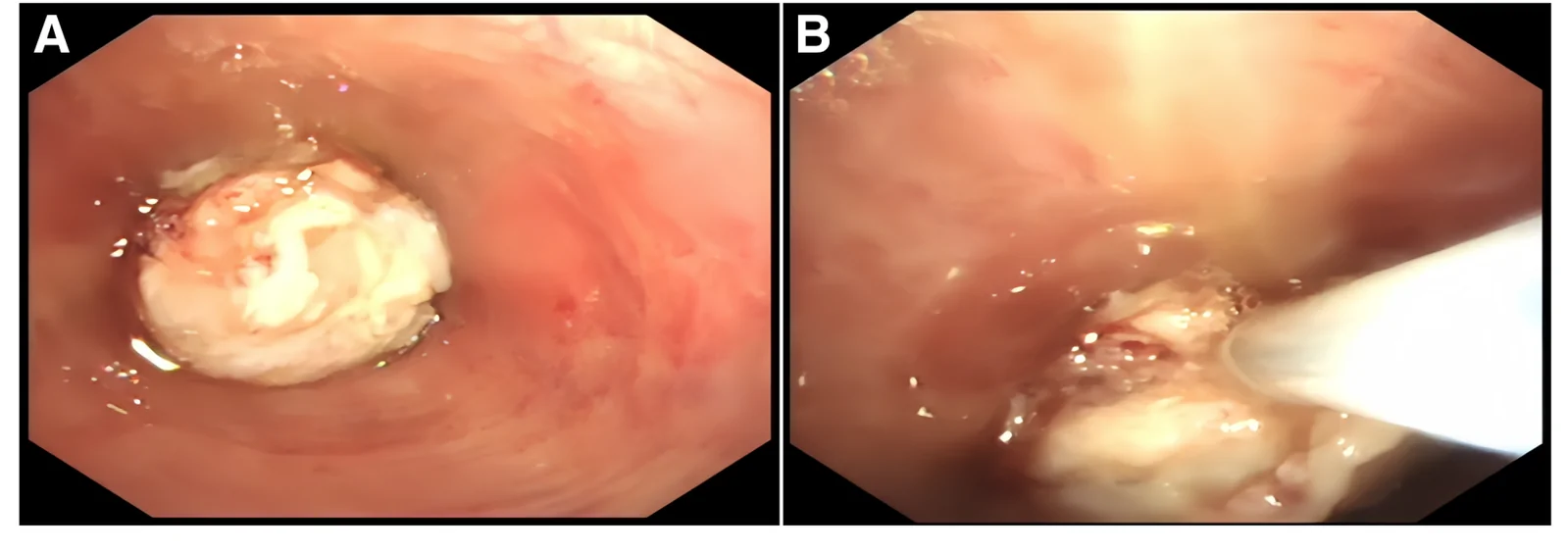
(A) A neoplasm obstructed the left main bronchus. (B) Endoscopic snare therapy was performed for the bronchial neoplasm.

On ECMO day 2, the patient underwent flexible bronchoscopy, which revealed a fleshy, highly vascular tumor completely obstructing the left main bronchus (Fig. 6A). Electrocautery snare was used to resect the obstructing lesion and obtain biopsy specimens for pathological analysis (Fig. 6B). During the procedure, significant bleeding occurred from the tumor bed, requiring emergent interventional radiology consultation. The patient was promptly transferred to the angiography suite, where bronchial artery embolization was performed – successfully controlling the hemorrhage. Notably, despite the absence of systemic anticoagulation and the presence of intraprocedural bleeding, no ECMO circuit thrombosis was observed throughout the course of support.

The patient was weaned from ECMO on day 3 and extubated concurrently, with stable oxygenation and no recurrent bleeding. Pathological examination of the biopsy specimens confirmed primary squamous cell carcinoma of the lung. Following recovery from the acute event, the patient was evaluated by thoracic surgeons and deemed a candidate for curative-intent surgery, given the absence of distant metastases. He underwent a successful left pneumonectomy 2 weeks later. At 2-week postoperative follow-up, he was recovering well, with normal oxygenation on room air, no respiratory compromise, and no evidence of recurrent bleeding or ECMO-related complications.

## 3. Literature review

The use of ECMO in patients with malignancy has evolved significantly over the past decade. Once considered a relative contraindication due to concerns about poor prognosis and heightened complication risks,^[13,14]^ ECMO is now increasingly utilized in select oncology patients – particularly for reversible causes of respiratory failure such as MCAO. Below, we review the contemporary literature focusing on 3 key domains: ECMO utilization for airway obstruction, anticoagulation strategies, and patient selection and outcomes.

### 3.1. ECMO for airway obstruction

A growing body of evidence – predominantly case series and small cohort studies – supports the use of VV-ECMO as a bridge to definitive airway intervention in patients with severe MCAO.^[17,18]^ A comprehensive 2025 review analyzed 27 studies encompassing 54 patients with tumor-related airway obstruction (caused by neck or chest malignancies) who underwent ECMO-supported airway interventions. The authors reported an 87% in-hospital survival rate, with only 7.4% of deaths attributed to disease progression unrelated to ECMO or procedural complications.^[16]^ This high survival rate underscores the utility of ECMO in addressing the immediate life threat posed by MCAO, even in patients with advanced malignancy. These findings are corroborated by a 2024 study, which described 29 consecutive patients with severe central airway obstruction (median tracheal diameter = 4.5 mm, interquartile range = 2–5 mm) who received VV-ECMO support for bronchoscopic or surgical interventions.^[18]^ Using a femoral–jugular cannulation approach and a minimal anticoagulation protocol (heparinized saline flushes only, no systemic heparin), all patients survived to 6-month follow-up, with minimal ECMO-related complications. This study highlights the safety and efficacy of VV-ECMO in even the most severe cases of airway obstruction, where conventional ventilation is impractical.

Among different modes of ECMO, VV-ECMO is used to support most airway surgeries or interventions, as it provides superior oxygenation to the upper body during prolonged apnea compared with veno-arterial ECMO.^[10,19,20]^ While data on ECMO for airway obstruction remain limited by small sample sizes and heterogeneity, current evidence suggests that elective VV-ECMO should be considered in patients with symptomatic MCAO (e.g., dyspnea, stridor, orthopnea) and a tracheal/bronchial diameter <5 mm (confirmed by CT or bronchoscopy),^[19,21]^ particularly when mechanical ventilation is ineffective or contraindicated.^[22]^

### 3.2. Anticoagulation strategies

Anticoagulation management represents one of the most significant challenges in ECMO-supported airway interventions. Traditional ECMO protocols require systemic anticoagulation to prevent circuit thrombosis, but this increases the risk of bleeding – especially during invasive procedures such as tumor debulking, which involves manipulation of highly vascular tissue.^[23]^ A large cohort study by Martucci et al of 652 VV-ECMO patients found that 77% received systemic anticoagulation, with over 50% experiencing at least 1 bleeding event – highlighting the inherent trade-off between thrombosis and bleeding risk.^[24]^ For cancer patients, this trade-off is particularly pronounced. A retrospective analysis by Kochanek et al of 297 cancer patients who underwent VV-ECMO across 19 European centers reported a 38% incidence of severe bleeding, which was independently associated with increased ECMO-related mortality (60-day survival rate 26.8% overall).^[15]^ These data underscore the need for individualized anticoagulation strategies in oncology patients undergoing ECMO-supported airway interventions.

Emerging evidence supports the safety of minimal or no systemic anticoagulation for brief ECMO runs (typically <72 h) in this setting. The 2024 Critical Care study referenced earlier employed a protocol of heparinized saline flushes (5 U/mL) without systemic heparin, with no cases of circuit thrombosis. Similarly, a 2025 case series described 10 patients undergoing ECMO-supported thoracic surgery, 6 of whom received no maintenance heparin – with no circuit failure due to thrombosis.^[25]^ A 2023 case report further demonstrated the feasibility of low-dose heparin during VV-ECMO for MCAO, reporting reduced intraoperative bleeding and shorter operative times compared to full therapeutic anticoagulation.^[26]^ Our case series aligns with these findings: all 3 patients received heparin-free VV-ECMO (circuit primed with heparinized saline only), with no circuit thrombosis observed. While case 3 experienced significant intraprocedural bleeding, this was successfully managed with bronchial artery embolization – emphasizing that bleeding risks in these patients are primarily related to tumor vascularity rather than anticoagulation status. Collectively, these data suggest that for short-duration ECMO support (e.g., for bridge to bronchoscopic intervention), minimal or heparin-free protocols are feasible and may be safer than full therapeutic anticoagulation, provided that close monitoring of circuit function and bleeding is performed.

### 3.3. Patient selection and outcomes

Appropriate patient selection appears crucial for successful outcomes when using ECMO as bridge therapy in oncology patients. A propensity score-matched analysis by Kochanek et al found no survival benefit of ECMO in cancer patients with irreversible respiratory failure.^[15]^ In contrast, outcomes appear more favorable when ECMO is used specifically as a bridge to intervention for reversible conditions like airway obstruction. The review mentioned before reported an 87% survival rate in this subgroup, reflecting the ability of ECMO to address the immediate life threat while allowing for subsequent anticancer therapy.^[16]^ This significant difference in outcomes based on indication highlights the importance of distinguishing between ECMO as salvage therapy for irreversible respiratory failure versus ECMO as a planned bridge to a specific intervention.

The importance of MDT decision-making in this process cannot be overstated – with input from intensivists, interventional pulmonologists, oncologists, thoracic surgeons, and perfusionists to balance risks and benefits. The American Society for Artificial Internal Organs published a case report in 2024 detailing successful bronchial stent placement under VV-ECMO for severe stenosis due to lung cancer. The authors emphasized the importance of multidisciplinary planning, with teams typically including intensivists, interventional pulmonologists, thoracic surgeons, perfusionists, and oncologists.^[27]^

## 4. Discussion

The 3 cases presented in this series, along with the supporting literature, demonstrate that VV-ECMO can serve as an effective bridge therapy to definitive interventions in selected patients with MCAO. This approach addresses a critical gap in the management of these complex patients, for whom conventional mechanical ventilation may be inadequate or even dangerous.

Our experiences align closely with findings from contemporary studies. First, the immediate procedural success in all 3 cases – with rapid restoration of oxygenation and airway patency – mirrors the 87% in-hospital survival rate reported in the 2025 review of 54 patients with tumor-related airway obstruction.^[16]^ This consistency underscores that ECMO effectively decouples gas exchange from native lung function, enabling safe performance of high-risk bronchoscopic interventions that would otherwise be precluded by the risk of intraprocedural asphyxiation.^[28]^ Notably, even in case 1 – where the patient ultimately died from progressive metastatic disease – ECMO successfully addressed the immediate life threat, providing meaningful symptomatic relief and allowing for a dignified discharge.

Second, our use of heparin-free VV-ECMO aligns with emerging evidence supporting minimal anticoagulation for brief ECMO runs. The 3 cases had ECMO durations of 23, 24, and 72 hours – consistent with the <72-hour window for which minimal or no anticoagulation has been shown to be safe.^[17,29,30]^ The absence of circuit thrombosis in all cases, despite the lack of systemic heparin, confirms that circuit priming with heparinized saline is sufficient to mitigate thrombosis risk in short-duration support. This is particularly relevant for oncology patients, who are at increased risk of bleeding due to tumor vascularity and potential thrombocytopenia or coagulopathy from prior anticancer therapy. Case 3, which involved significant intraprocedural bleeding managed successfully with bronchial artery embolization, highlights the importance of avoiding unnecessary systemic anticoagulation in this population – while also emphasizing the need for immediate access to interventional radiology as a contingency for bleeding complications.

Third, the critical role of MDT collaboration is evident across all 3 cases. From the initial decision to initiate ECMO to procedural planning (e.g., cannulation approach, bronchoscopic technique) to post-intervention care (e.g., weaning from ECMO, management of infection or bleeding), the MDT ensured a coordinated, patient-centered approach. This aligns with the literature, which consistently emphasizes MDT involvement as a key determinant of optimal outcomes in ECMO-supported oncology patients. In particular, the integration of interventional radiologists in case 3 was pivotal to managing the acute bleeding complication – underscoring the need for a comprehensive team with diverse expertise.

Several additional considerations emerge from our analysis, which may inform clinical practice. First, the choice of VV-ECMO over veno-arterial ECMO is justified in these cases, as all patients had isolated respiratory failure without hemodynamic compromise. VV-ECMO provides superior upper body oxygenation during apnea – critical for maintaining cerebral perfusion during bronchoscopic interventions that require temporary airway manipulation.^[10,29]^ Second, pre-procedural imaging (e.g., chest CT, CT angiography) is essential to assess both airway anatomy (to plan the intervention) and vascular anatomy (to optimize cannulation and avoid complications such as vessel injury).

This study has several limitations that warrant acknowledgment. First, the small sample size (3 cases) limits the generalizability of our findings, and selection bias cannot be excluded – given that these patients were carefully selected by the MDT for ECMO support. Second, the retrospective nature of case series design precludes causal inference, and larger prospective cohort studies or randomized controlled trials are needed to validate these findings. However, given the rarity and urgency of MCAO, such trials are logistically challenging – making case series and systematic reviews the primary sources of evidence in this field. Third, we did not assess long-term quality of life or functional outcomes, which are important considerations in palliative interventions for advanced malignancy.

Future research should focus on developing more precise patient selection criteria, optimizing anticoagulation protocols for brief ECMO runs, and evaluating long-term outcomes beyond procedural success. Comparative studies between different management strategies for malignant airway obstruction would be particularly valuable, though challenging given the relative rarity and urgency of these cases.

In conclusion, VV-ECMO represents a viable and potentially life-saving bridge to definitive interventions in carefully selected patients with MCAO. When implemented by experienced MDTs with appropriate patient selection and individualized management, this approach can successfully address an otherwise fatal complication of thoracic malignancies, providing both immediate life-saving support and the opportunity for subsequent anticancer therapies.

## Acknowledgments

The authors thank the patients for consenting to publication of this report.

## Author contributions

**Data curation:** Siyu Yang, Qing Liu, Lan Ni.

**Formal analysis:** Siyu Yang, Qing Liu, Lan Ni.

**Investigation:** Siyu Yang, Chaojie Wei.

**Project administration:** Siyu Yang, Zhenshun Cheng, Chaojie Wei.

**Conceptualization:** Zhenshun Cheng, Chaojie Wei.

**Supervision:** Zhenshun Cheng, Chaojie Wei.

**Funding acquisition:** Chaojie Wei.

**Resources:** Chaojie Wei.

**Writing – original draft:** Siyu Yang, Chaojie Wei.

**Writing – review & editing:** Siyu Yang, Zhenshun Cheng, Chaojie Wei.
